# Eco-Geopolymers: Physico-Mechanical Features, Radiation Absorption Properties, and Mathematical Model

**DOI:** 10.3390/polym14020262

**Published:** 2022-01-09

**Authors:** Neslihan Doğan-Sağlamtimur, Ahmet Bilgil, Sefa Ertürk, Vakkas Bozkurt, Elif Süzgeç, Arife Gözde Akan, Pervin Nas, Hüseyin Çetin, Magdalena Szechyńska-Hebda, Marek Hebda

**Affiliations:** 1 Department of Environmental Engineering, Niğde Ömer Halisdemir University, Nigde 51240, Turkey; elfszgc591@gmail.com (E.S.); g.akan96@gmail.com (A.G.A.); nas_pervin01@hotmail.com (P.N.); 2 Department of Civil Engineering, Niğde Ömer Halisdemir University, Nigde 51240, Turkey; abilgil@ohu.edu.tr (A.B.); hcetin@ohu.edu.tr (H.Ç.); 3 Department of Physics, Niğde Ömer Halisdemir University, Nigde 51240, Turkey; sefaerturk@gmail.com (S.E.); vakkasbozkurt@ohu.edu.tr (V.B.); 4 The Plant Breeding and Acclimatization Institute—National Research Institute, Radzików, 05-870 Błonie, Poland; szechynska@wp.pl; 5 Faculty of Materials Engineering and Physics, Cracow University of Technology, Warszawska 24, 31-155 Krakow, Poland; marek.hebda@pk.edu.pl

**Keywords:** environment, fly ash, geopolymer, building material, radiation absorption, waste reuse

## Abstract

Waste ashes and radiation are hazardous environmental and health factors; thus, a lot of attention is paid to their reduction. We present eco-geopolymer building materials (GPBMs) based on the class F fly ashes (FFAs) from thermal power plants (TPPs) and their implementation as a barrier against radioactive radiation. Different methods of production, ratios of FFA to alkali activator, and temperatures of curing were tested. Small spherical particles and higher content of SiO_2_ resulted in developed surface area and higher reactivity of Isken TPP FFA than Catalagzi TPP FFA. Lower activator concentration (10% vs. 20%) and curing temperature (70 vs. 100 °C) caused an increase in GPBM compressive strength; the highest value was measured as 93.3 MPa. The highest RA was measured for GPBMs, provided alkali activator ratio (Na_2_SiO_3_/NaOH) was >2 and its concentration was 20%. The mathematical model developed in this study proved FFA quantity, and thus GPBM mechanical properties, as key factors influencing RA. In the light of these results, the lightweight GPBMs can be excellent materials for the construction sector dedicated to immobilization, storage, and disposal for radionuclides or barriers against radiation; however, multiple steps of their production require careful optimization.

## 1. Introduction

The demand for building materials is increasing every day; currently, the global consumption of concrete is ranked second after water. The production of ordinary Portland cement (OPC) consumes both energy and natural resources. As stated in the International Energy Agency’s Greenhouse Gas R&D Programme, cement production releases approximately 50% of total CO_2_ emission (0.81 kg CO_2_ per kg cement) [1]. 

Geopolymers have been studied extensively as an alternative building material that is environmentally friendly and serves as part of sustainable development [2,3,4]. Comparing the OPC, the main advantage of a geopolymer is outstanding mechanical properties and durability [5,6,7,8], chemical resistance [3], and thermal resistance [9]. Moreover, geopolymer production is cost-effective and has a 60–80% lower carbon footprint and resource consumption. Existing studies usually focus on ecological aspects of geopolymers, including synthesis of geopolymers from waste and disposal of radioactive waste in the geopolymer matrix [9,10,11]. Geopolymers are also novel in the context of removing organic pollutants from water and air to protect and improve the environment [12,13].

Davidovits introduced the term “geopolymers” to assign a new class of aluminosilicate materials with a three-dimensional structure, and he developed alkali activated fly ash (FA)-based geopolymers [14]. FA is an industrial solid waste released from coal-fired power plants. FA is produced from the burning of pulverized coal in a coal-fired boiler and is usually collected from the flue gas by electrostatic precipitators or mechanical collection devices such as cyclones. When pulverized coal is combusted in a dry-bottom boiler, about 80% of the ash leaves the furnace as FA in the flue gas. When pulverized coal is combusted in a wet-bottom (or slag-tap) furnace, 50% of the ash is retained in the furnace, with the other 50% being entrained in the flue gas. During this process, some parts of coal cannot be ignited or burned; thus, FA includes combustible material. FA has a chemical composition that is difficult to control, and the quality of FA depends on the type of coal and efficiency of the power plant. FA usually comprises fine particles and a high amount of amorphous silica and alumina; therefore, it is used as a raw material to produce geopolymers [15,16]. High strength geopolymers are produced based on a class F FA [17,18]. To obtain alumina and silica precursors, FA should be mixed with an alkali solution [19,20]. Common alkali activators are sodium hydroxide (NaOH), potassium hydroxide (KOH), or their combination, used together with sodium silicate (Na_2_SiO_3_) or potassium silicate (K_2_SiO_3_) [21]. 

The final properties of a geopolymer depend on many variables concerning raw materials, i.e., (1) mineralogical and chemical composition, (2) structural properties, and (3) physical properties. Similarly, many factors affect the properties of the geopolymer during the production process; (4) ratio of raw materials, (5) duration of mixing and temperature of the mixture, (6) rheology modifiers, accelerators, or retarders influencing the setting time, and (7) curing time. As the design and the appropriate selection of ingredients in laboratory conditions are costly and time-consuming, to achieve the mechanical properties supporting the usage of FA in the building industry, research using data-driven methods, artificial intelligence, machine learning methods, and precise mathematical modeling should be developed [21]. 

Much research has been performed to assess geopolymer materials as a matrix for immobilization, storage, and disposal of radionuclides or as barriers against radiation. Different types of harmful radiation, i.e., neutron, X-ray, and gamma-ray can penetrate the conventional cement walls and roofs and easily escape from accelerators, hospitals, and nuclear power plants. If required, heavy concrete (approximately 1.5 times heavier and denser than OPC, with density 2900–6000 kg m^−3^) is used for wall and roof barriers [22]; however, interest in the production of the geopolymer barriers that absorb radiation more efficiently than concrete has gradually increased [11,23,24,25,26]. A number of studies investigating geopolymer barriers focus on their behavior in the context of radioactive element leaching (e.g., cesium (Cs), strontium (Sr)). In this case, the selection of a precursor material and appropriate activator has a key impact due to immobilization occurring via ion exchange, physical adsorption, and encapsulation by the gel matrix [11]. The FA-based geopolymer showed an excellent immobilization of Cs and Sr, while slag-blended geopolymers and OPC were less suitable [27]. Sodium-based geopolymers had a better selectivity for Cs at a molar ratio Cs/Al = 0.3 and Na/Al ratio range from 0.7 to 1.3. Sr leaching was greater in acidic conditions than in deionized water [26]. Radioactive borate waste was solidified using metakaolin activated with KOH [28]. Low-Ca geopolymers had a unique ability to immobilize nuclide ions, due to the cross-linked network [11]. In the case of the geopolymer barrier, the effect of barite in FA-based geopolymer was also investigated [22]. The study showed that the attenuation coefficient was directly related to the type of raw material, but there was no significant effect of change in its aggregation on the radiation absorption (RA) properties of the geopolymer. Furthermore, studies were performed to design a shielding material based on a geopolymer material with 15% barium sulphate [29] and heavyweight aggregates [30,31]. Due to the excellent immobilization of low- and intermedium-level radionuclide waste, geopolymers were approved for a waste package for sludge/resin mixture by the Slovak and Czech Nuclear Regulatory Authorities (i.e., the SIAL^®^ matrix). Approximately 29.6 m^3^ (38.15 t) of waste with total activity around 4.94 × 10^12^ Bq were immobilized between 2003 and 2010 [26,32].

In most countries, the vast majority of FA is stored in authorized warehouse sites. The limited possibilities of the usage of class F fly ash (FFA) necessitate efforts to solve the environmental and economic problems with waste. In terms of the number of power plants, Turkey ranks near the top 15 in the world [33]. According to Özkan et al. [34], the annual amount of FA exceeded 50 Mt in 2020. In this study, eco-geopolymer building materials (GPBMs) were produced based on FFAs from Catalagzi Thermal Power Plant (TPP) (Zonguldak, Turkey) and Isken Sugozu TPP (Adana, Turkey) as raw materials and NaOH and/or Na_2_SiO_3_ as alkali activators. The RA percentage of GPBMs was measured using a Cs-137 radiation source. In addition, mathematical model was developed to predict RA instead of conducting time-consuming and costly experiments. The experimental data were fitted to a polynomial function using the least-squares method. The polynomial functions were maximized under the substance constraints to find optimum mixtures and achieve the maximum RA (%). The study intended to reduce the environmental problem, contribute to a circular economy and zero waste principles by means of waste FFA reuse as a raw material, and reduce carbon footprint and global warming by using cementless geopolymer technology and GPBMs with a high capacity of RA (comparing to conventional building materials). Therefore, following the definition of “eco-materials” in the Eco-products Directory 2010, geopolymers developed in this study refer to “materials (or material technologies) that possess excellent characteristics with good performance, which can be manufactured, used, and recycled or disposed of, while having only a low impact on the environment as well as being kind to humans”. With the above in mind, we described developed materials as eco-geopolymer.

## 2. Materials and Methods

### 2.1. Raw Materials Characteristic

Morphology of FFAs supplied from Catalagzi and Isken TPPs was determined with a Zeiss/EVO 40 scanning electron microscope (Jena, Germany). Sieve analysis (UTEST UGT0411, Ankara, Turkey) of the FFAs was performed according to the Turkish Standards—European Norm (TS EN) 933-10 [35]. The chemical composition of the FFAs was analyzed with an X-ray fluorescence (XRF) spectrometer (Zetium-X, PANalytical. B.V, Almelo, Netherlands), equipped with an ultra-high performance X-ray tube with a 2.4 kW rhodium anode. To assess the amount of residual combustible material, 20 g of FFA was oven-dried at 105 °C, and the sample was weighed (x), kept 2 h in a muffle furnace (Kaleo RS150, Kutahya, Turkey) at 750 °C, then cooled to room temperature and weighed (y). Loss on ignition (LOI) was calculated by the formula: [(x – y)/x] × 100. The specific surface area; micro-, meso-, and macro-pore size; as well as distribution of the pore size were determined as a function of relative pressure with the Brunauer–Emmett–Teller (BET) method using a Quantachrome/NOVAtouch LX4 (Anton Paar, Graz, Austria) physical sorption analyzer. The sample degassing temperature was 300 °C, rate 20 °C min^−1^, soak time 180 min, and relative pressure (p/p0) in the range from 0.021 to 0.994 for 44 measuring points. The results were analyzed using the AsiQwin software (Anton Paar, Graz, Austria).

### 2.2. GPBM Assessment Methods

GPBMs were produced based on FFAs and the RILEM Cembureau standard sand [36]. Alkali activators consisted of 12 M NaOH (97% purity) and Na_2_SiO_3_ (29.4% SiO_2_, 14.7% Na_2_O, and 55.9% H_2_O, by mass). Alkali activator NaOH was used at a ratio of 10%, 15%, 25% alkaline activator to FFA (by weight); or NaOH/Na_2_SiO_3_ solutions (1:0, 1:1, 1:1.5, 1:2, and 1:2.5) were used at a ratio of 10% and 20% alkaline activator to FFA (by weight) (Table 1). The samples were selected according to their mechanical properties for further studies.

To produce GPBMs based on FFA (without the RILEM Cembureau standard sand), “separate” and “normal” mixing methods were tested. For “separate” mixing, FFA was mixed with NaOH for 10 min to enable the leaching of ions. Although, the paste was relatively viscous, subsequently added Na_2_SiO_3_ solution required short mixing to obtain a homogeneous paste. For “normal” mixing, FFA, NaOH, and Na_2_SiO_3_ were mixed for 1 min [14]. To produce GPBMs based on FFA and the RILEM Cembureau standard sand, all ingredients were mixed approximately for 3 min. Paste workability was determined in terms of flow characteristics according to the ASTM C1437-15 [37]. The pastes were poured into a mold with dimensions of 4 × 4 × 16 cm. GPBMs were cured 24 h at 70 and 100 °C. After completing the thermal curing, the samples were demolded and kept at room temperature until the testing time at 7, 28, and 90 days.

GPBM density was calculated according to the following equation (Equation (1))

γ = m_ad_/V_g_ (g/cm^3^), (1)

where m_ad_ is the air-dried mass, and V_g_ is the gross volume of the samples.

The water absorption of the samples was analyzed according to TS EN 772-3 [38]. Water absorption by mass (Am) and by volume (Aw) was calculated according to the following equations (Equations (2) and (3))

Am = (m_sa_ − m_d_)/m_d_ ⋯ 100 (%), (2)

where m_sa_ is the saturated mass and m_d_ is the dried mass

Aw = (m_sa_ − m_d_)/(m_sa_ − m_s_) ⋯ 100 (%), (3)

where m_s_ is the sample mass (according to the Archimedes principle).

The porosity was a measure of “empty” spaces in a material and was expressed as a volume of voids relatively to the total volume (apparent porosity).

The flexural strength test was performed according to the ASTM C348-14 [39] and the compressive strength test according to the ASTM C349-14 [40].

**Figure 1:**
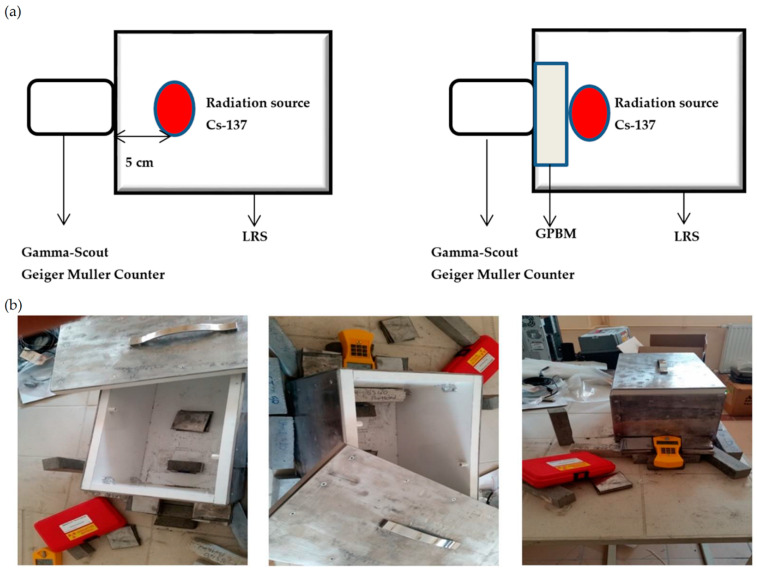
Experimental setup: (**a**) scheme of RA measurement and (**b**) a newly designed LRS cage.

RA measurements were performed using a Geiger–Müller counter and Cs-137, a source of gamma rays of high energy. The scheme of the experimental setup is given in Figure 1a. A lead radiation shield (LRS) cage with a lattice system consisting of nested lead plates (4 mm thick) was designed and applied to avoid uncontrolled radiation (Figure 1b).

In the first step, gamma rays emitted from the radioactive source were measured in the absence of GPBMs (Figure 1a). In the second step, GPBM was placed between radioactive source and Geiger–Müller counter (Figure 1a). In both steps, data were recorded for one hour. The radiation absorbed by the sample was calculated as a ratio from both measurements.

In accordance with TS EN 12390-3 [41], all experiments were performed with at least three repetitions. The repetition (*n*) was defined as an individual sample at given conditions. The repeatability of the result of measurement was below 9%.

## 3. Results and Discussion

### 3.1. The Properties of Raw Materials

FFAs are considered as important raw materials for geopolymer production; however, their reactivity depends on chemical and physical properties. To prove the potential of the Catalagzi TPP FFA and the Isken TPP FFA for utilization in the geopolymerization process, the chemical composition was analyzed (Table 2). The amounts of SiO_2_ and Al_2_O_3_, the most important determinants of the geopolymerization process, were relatively high in both the Catalagzi TPP FFA and the Isken TPP FFA. Acid oxides SiO_2_ and Al_2_O_3_, occurring mainly in the glassy phase, show high reactivity in an alkaline environment [10,42]. Due to such properties, construction GPBM products can be formed on the basis of the Catalagzi TPP FFA and the Isken TPP FFA. However, comparing both FFAs, the Isken TPP FFA had a higher content of silica + alumina (80.16% and 83.74% in the Catalagzi and Isken TPP FFAs, respectively). Bearing in mind that the geopolymer material became increasingly elastic with the increasing total SiO_2_ content in the raw materials, and a compressive strength increases along with the Si/Al ratio [13,21], higher potential of Isken TPP FFA for the geopolymerization process was expected. The ratio of SiO_2_/Al_2_O_3_ was 2.07 and 2.90. Therefore, geopolymerization could be favored in forming a poly(silate-siloxo) (-Si-O-Al-O-Si-O-) structure in which the Si/Al ratio is 2 and a poly(silate-disiloxo) (-Si-O-Al-O-Si-O-Si-O) structure in which the Si/Al ratio is 3, rather than poly(silate) (-Si-O-Al-O-) in which the Si/Al ratio is 1 [21]. The CaO amount was less than 10% in both FFAs; however, the amount of CaO together with MgO was higher in the Catalagzi TPP FFA than Isken TPP FFA. The impact of calcium on the geopolymer is usually positive; a composite system with geopolymer gel and calcium-silicate-hydrate gel can coexist when the calcium content increases. Therefore, the final geopolymer properties may be the complementary result of both factors—the total content of Si and Al components but also the modifying effect of CaO [21]. FFAs can be classified as; (i) silica-aluminum, (ii) alumina-silica, (iii) calcium sulphate, and (iv) calcium types [43,44]. According to the results, both FFAs can be defined as an alumina-silica type. The FFA classification according to the ASTM C618 standard distinguishes the FFA class F or C. Basically, F class is silica ash obtained from the combustion of bituminous coal (hard and/or brown coal). Class C ashes are rich in calcium oxide and result from the combustion of sub-bituminous coals and lignite (brown coal). Additionally, F class FAs are characterized by the sum of SiO_2_ + Al_2_O_3_ + Fe_2_O_3_ components higher than 70%, while for class C FAs this ratio is higher than 50 (by weight) (ASTM C618) [45]. The chemical composition of both the Catalagzi and Isken TPP FFAs confirmed the classification as the F type FAs.

LOI (%), an indicator of residual carbon content, is a critical parameter when evaluating the effectiveness of the geopolymerization process. LOI values were determined as 1.52% and 1.78% for the Catalagzi TPP FFA and Isken TPP FFA, respectively (Table 2). Although obtained LOI values are common (e.g., [21,46]), it is worth noticing that the residual carbon from an inefficient coal combustion process in the power plant can absorb water and chemical admixtures. Further, it results in a change of air-void system in the geopolymer, negatively affects production of building materials, and reduces their frost resistance. The LOI values obtained for the Catalagzi and Isken TPP FFAs were relatively low, confirming that the Catalagzi and Isken TPP FFAs were suitable raw materials for the high-efficiency geopolymerization and GPBM production.

**Figure 2:**
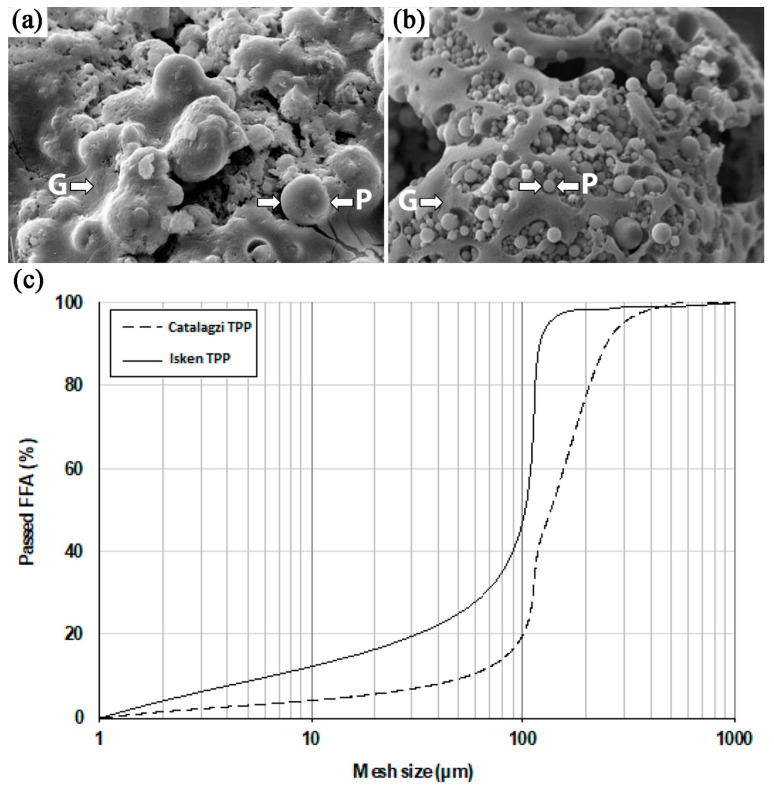
Morphology of (**a**) the Catalagzi TPP FFA, (**b**) the Isken TPP FFA, and (**c**) particle distribution.

Different chemical composition was related to a variance in particle morphology of the Catalagzi TPP FFA and the Isken TPP FFA (Figure 2a,b). Generally, the particle morphology of both FFAs (Figure 2) indicated that they came from conventional dust furnaces, in which the temperature ranged from 1200 to 1600 °C, and finely ground coal was used. The high temperature of combustion created a spherical shape of particles and a glassy phase. The particles of FFA with spherical morphology are beneficial in order to achieve a successful polymerization process as they improve the rheological properties of the paste, increasing its workability [21]. Both FFAs had spherical particles (Figure 2a,b). However, more random particle geometry, rough and porous surface texture, and tendency to form agglomerates intermixed with the glassy phase were found for the Catalagzi TPP FFA. In contrast, a fraction of individual, almost perfectly spherical balls, clearly separated from the glassy phase, was observed for the Isken TPP FFA. Both FFAs had particles with a maximum size of 500 µm; however, the particle frequency of the Isken TPP FFA with a size smaller than 100 µm was twice as high when compared to the Catalagzi TPP FFA (Figure 2c). The phenomenon is attributable to the greater surface area available for chemical reactions. The smaller particles have a larger surface area in comparison to the volume and thus, higher reactivity, including the rate of dissolution of the monomers, i.e., silicate and aluminate, consequently resulting in a more effective geopolymerization process [21].

Differences in particle morphology were confirmed by physical properties, i.e., the specific surface area of FFAs. BET values were determined as 1.11 for the Catalagzi TPP FFA, and two-fold higher values of 2.26 m^2^ g^−1^ were measured for Isken TPP FA (Table 3). The mean specific gravity of coal ashes was around 2.0. The values were standard; however, a variation between Catalagzi TPP FFA and Isken TPP FFA was resulted from a combination of several factors, such as particle shape, distribution, and chemical composition [47]. Along with a decrease in the size of particles, the density of the geopolymer increased. Indeed, in all cases higher values of air- and oven-dried and loose and tight bulk density (Table 3) were calculated for the Isken TPP FFA than Catalagzi TPP FFA; the smaller particles of Isken TPP FFA filled space in a more compact way.

The raw materials consisted of the RILEM Cembureau standard sand (Table 4) which complies with the TS EN 196-1 specification [48]. FFA and standard sand are two different materials. Indeed, the RILEM Cembureau standard sand has a specific gravity of 2.56 and density of 1.35 kg dm^−3^ which are significantly higher than those measured for FFAs. Therefore, an addition of sand can decrease the total surface area of granular ingredients affecting the water demand and paste workability, hydration rates, and strength of geopolymers.

Considering the chemical and physical properties, the construction GPBM products can be formed on the basis of the Catalagzi TPP FFA and the Isken TPP FFA as well as with the addition of the RILEM Cembureau standard sand.

### 3.2. GPBMs Production and Properties

In this study, FFAs were activated through grinding, heating, and then alkali solutions. To produce the GPBM samples, different methods of sample preparation were compared. Sequentially added ingredients required double mixing with a total time of almost 12 min. In the first step, paste consisting of FFA and NaOH was relatively viscous. Liquid Na_2_SiO_3_ was added in the second step. Another protocol consisted of FFA, NaOH, and Na_2_SiO_3_ mixing for 1 min to obtain homozygous mortar. It was previously shown that length of mixing period and mortar viscosity affect geopolymer setting time [21]. The water in the mixing phase determines the workability of the paste during the production of the geopolymer. However, water evaporating from the geopolymer during the curing process creates discontinuous nanovoids. Therefore, with a shorter setting time (when the lower amount of free liquid is present) and improved homogeneity of mortar, a lower number of voids can occur, and higher compressive strength of the geopolymer can be achieved. Indeed, a “normal” mixing procedure resulted in a geopolymer product with a higher strength compared to the geopolymer produced by the separate mixing method. Therefore, a normal mixing procedure was preferred in further studies. On the other hand, discontinuous nanovoids were present also in materials prepared according to the “normal” mixing protocol and resulted in lightweight GPBMs with a density below 2 g cm^−3^.

Furthermore, FFA type, activator ratio (NaOH and/or Na_2_SiO_3_), and curing temperature were the key factors modifying geopolymer porosity (Table 5). For the Catalagzi TPP FFA produced at higher curing temperature (100 °C), the GPBM densities were generally lower. In the case of the activator ratio, a higher NaOH content vs. Na_2_SiO_3_ content and lower geopolymer porosity were observed. The lower porosity correlated with lower water absorption of the final GPBM products.

Generally, the raw material reactivity was controlled by numerous intrinsic (e.g., the mineralogical, chemical, structural composition, amount of reactive fraction of SiO_2_ and Al_2_O_3_) and extrinsic parameters (e.g., FA fineness level, type and proportion of raw materials, the ratio of activators, duration and temperature of the process) (Figure 1, Table 2, Table 3, and Table 5), which consequently affected GPBM mechanical properties and homogeneity (Table 5). It is well known that a greater fineness level of the FFA increases paste workability and the rate of geopolymerization, shortens setting time, and improves physical (density), mechanical (compressive and flexural strength), and microstructural (compact and uniform matrix) properties of the geopolymer [21,44]. The mechanism of improving the physical and mechanical properties of GPBM is also attributed to a higher amount of alkaline aluminosilicate gel [21,49,50,51,52]. Accordingly, the better mechanical properties of the geopolymer were obtained when the Isken TPP FFA was used as raw material (Figure 2, Table 2 and Table 3). Flexural strength, also known as modulus of rupture, or bending strength, or transverse rupture strength, is a material property defined as the stress in a material just before it yields in a flexure test. The flexural strength was dependent only on FFA type, and higher values were found for the GPBM samples prepared from the Isken TPP FFA (Table 5). The compressive strength test is a mechanical test measuring the maximum amount of compressive load a material can bear before fracturing. Compressive strength is the most used criterion in engineering applications because is of vital importance for the structural integrity of building materials in both the construction phase and after the construction is completed [53,54]. In this study, GPBMs with the best compressive strength values (>30 MPa; the GPBMs presented in Table 5) were selected among 169 produced materials, and similarly, as in the case of flexural strength, GPBMs produced from the Isken TPP FFA had higher compressive strength values. The range of compressive strength was in agreement with earlier studies [55]; however, it is worth noting that lower activator concentration (Na_2_SiO_3_/NaOH = 2, 10%) and lower curing temperature (70 °C) caused an increase in compressive strength of GPBMs, in comparison to a higher temperature (100 °C). The opposite effect was found for the higher amount of activator (Na_2_SiO_3_/NaOH = 2, i.e., 20%); a decrease in the curing temperature (70 °C) caused a decrease in compressive strength of GPBMs.

Radiation causes serious harm to the environment and humans; thus, effective shielding from radiation sources should be provided. Even though the determination of the RA is important, there is a limited number of studies concerning this parameter in GPBM. In this study, the RA test was performed in a newly designed LRS cage according to the ASTM and TS EN standards. The RA of conventional OPC was 9.52%, while GPBMs produced in different combinations of ingredients, mixing ratios, and curing temperatures had higher RA percentage values (Table 5). The highest RA of 12.54% was found for GPBMs produced from the Isken TPP FFA by activation with Na_2_SiO_3_/NaOH at a ratio of 2.5 and concentration of 20% (Table 5, sample number 22). Generally, the GPBMs produced from the Catalagzi TPP FFA had lower RA values; among them, the highest RA values were measured in alkali activator ratio of 2.0 (Na_2_SiO_3_ to NaOH) and its concentration was 20% (Table 5, sample number 2). Taking into account that: (1) geopolymers have good (or better) mechanical and chemical characteristics such as compressive strength, resistance to high temperature, acid resistance, and capacity to immobilize toxic elements (when compared to OPC) [28,56,57]; (2) GPBMs with the highest RA capacity also had a high density, flexural strength, compressive strength, and less porosity (Table 5); and (3) GPBMs produced from the Isken TPP FFA had higher RA and, at the same time, better mechanical and physical parameters than GPBMs produced from Catalagzi TPP FFA (Table 5), GPBMs proved suitable as an alternative material for a barrier against radiation. However, the properties which are suitable for providing the production process are optimized based on precise chemical analysis of the raw material, establishment of appropriate alkaline activators, and temperature of the process. Particularly, optimization should include the impact of these factors on structural continuity/porosity of the final product. It is an important issue in the context of direct functionality of geopolymer barriers as well as in the case of degradation in geopolymer material over time or in a dose-dependent manner. Little is known about the geopolymer performance under radiation; however, some studies suggest that direct changes in the pore structure due to radiation were found to be minimal. However, dose rate effect was observed in case of H_2_ generation from water by the transfer of absorbed energy from the solid geopolymer to the pore water [28,58]. Thus, the additive effect of changes in air–water status of geopolymer material and changes in the temperature of the external environment should be taken into account.

### 3.3. Optimization Methods, Curve Fitting, and Mathematical Modeling

Bearing in mind the importance of factors described above, such as chemical composition of the raw materials, ratio of ingredients, and curing temperature, it is necessary to have a tool that enables the selection of individual parameters and results in maximum RA values for geopolymer barriers against radiation. Mathematical modeling and appropriate algorithms can provide this without the need for costly experimentation.

Deviations during experiments are inevitable, but extreme deviations of individual measurement points should be eliminated. The least-squares method overcomes the deviation in experimental data. This technique results in a fitted curve. In order to find an optimally fitted curve, the data points and fitted curve can be denoted by (*x_i_*, *y_i_*) and *q*(*x_i_*) (*i* = 1, 2, 3… *n*). The i^th^ point error *ε_i_* which gives the difference between *y_i_* and *q*(*x_i_*) (*i* = 1, 2, 3… *n*) can be described by the following equation:

(4) $$\varepsilon_{i}=y_{i}-q(x_{i})$$

If $q(x_{i})$ is defined as the polynomial function, the total error can be expressed as follows:

(5) $$\sum_{i}^{N}\varepsilon_{i}=\sum_{i}^{N}\left(\;y_{i}-q(x_{i}\right))$$

(6) $$\sum_{i}^{N}\varepsilon_{i}=\sum_{i}^{N}\left(\;y_{i}-\left(\alpha_{0}+\alpha_{1}x_{i}+\alpha_{2}x_{i}^{2}+\cdots +\alpha_{n}x_{i}^{n}\right)\right)$$

In order to obtain an optimally fitted curve for the data set, the square of the total error for points E should be at a minimum, and this can be described as:

(7) $$E=\sum_{i}^{N}{\left(\varepsilon_{i}\right)}^{2}=\sum_{i}^{N}{\left(\;y_{i}-\left(\alpha_{0}+\alpha_{1}x_{i}+\alpha_{2}x_{i}^{2}+\cdots +\alpha_{n}x_{i}^{n}\right)\right)}^{2}$$

Therefore, each partial derivative for each indefinite coefficient of E needs to be zero, and this can be expressed as:

(8) $$\frac{\partial E}{\partial \alpha_{0}}=0=-2\sum_{i}^{n}\left(\;y_{i}-\left(\alpha_{0}+\alpha_{1}x_{i}+\alpha_{2}x_{i}^{2}+\cdots +\alpha_{n}x_{i}^{n}\right)\right)$$

(9) $$\frac{\partial E}{\partial \alpha_{1}}=0=-2\sum_{i}^{n}\left(y_{i}-\left(\alpha_{0}+\alpha_{1}x_{i}+\alpha_{2}x_{i}^{2}+\cdots +\alpha_{n}x_{i}^{n}\right)\right)x_{i}$$

(10) $$\frac{\partial E}{\partial \alpha_{n}}=0=-2\sum_{i}^{n}\left(y_{i}-\left(\alpha_{0}+\alpha_{1}x_{i}+\alpha_{2}x_{i}^{2}+\cdots +\alpha_{n}x_{i}^{n}\right)\right)x_{i}^{n}$$

If the equalities above are arranged for an *n*th degree curve, the (*n* + 1)th particle, the curve, and undefined coefficients can be calculated [59] with the equation as given below:

(11) $$\sum_{n=1}^{n}q(x_{i})\cdot \frac{\;\partial q(x_{i})}{\partial \alpha_{k}}=\sum_{n=1}^{n}y_{i}\cdot \frac{\;\partial q(x_{i})}{\partial \alpha_{k}}$$

The basis of equations in this study is related to the working principle of the least-squares fit method considering linear combinations of functions of the variables derived from experimental data. The importance of the mathematical model is to facilitate the prediction of RA % without the need for experiments. Furthermore, due to the optimization of the mathematical model, a mixture design with a maximum RA % was obtained.

Numerical algorithms for optimization of nonlinear constraint can be divided into two methods, namely the gradient-based method and direct search method. Whereas the gradient-based optimization method depends on the first and second derivatives, the direct search method does not use the derivatives. The convergence of the direct search method is slower than the convergence of the gradient-based method, but the former method is more robust and has higher tolerance to noise in the objective function and constraints. In this study, two different numerical direct search methods were used, namely the simulated annealing (SA) and the differential evolution (DE) algorithms [60].

The SA algorithm is a random optimization method and simple stochastic function minimizer. The method is based on the terminology and genesis of the annealing process, i.e., a feature related to the temperature variation to be embedded in the operational characteristics of the algorithm. Using optimization terminology, annealing allows the structure to escape from a local minimum, explore, and settle on a minimum. Due to the annealing procedure, the optimum point avoids the local point and allows the global optimum of a given function to be approximated. The objective function in the DE method is defined under equality or inequality constraints. In the first step, the initial population determined with the target vector and donor vector is created by a mutation. To define the test vector for the new generation, the target vector and donor vectors are compared with the purpose of choosing the lower one. Therefore, if the test vector is lower than the target vector, the optimization algorithm is finished, else the procedure returns to the mutation step to produce a new generation.

Considering the results of the experiments (Table 5), optimum curves were fitted as multivariable polynomials functions for RA (%). The curves were fitted to polynomial functions, using the command “Fit” in the Wolfram Mathematica 11 program. As a result of the curve fitting method based on the least-squares method, the equations 12 to 15 were derived using the data set from Table 5. RA was described by the following equations:

for GPBMs produced from the Catalagzi TPP FFA at 70 °C

(12) $$\mathrm{RA}\left(\%\right)=\left(6.06681\times 10^{-13}\right)a^{5}+\left(1.912\times 10^{-10}\right)a^{4}+\left(5.96302\times 10^{-8}\right)a^{3}+\left(1.677\times 10^{-5}\right)a^{2}+\;\left(3.0725\times 10^{-3}\right)a+\left(9.97403\times 10^{-13}\right)b^{5}-\left(1.32288\times 10^{-15}\right)c^{5}-\left(1.85411\times 10^{-15}\right)d^{5}+\;\left(2.70814\times 10^{-10}\right)b^{4}-\left(1.17738\times 10^{-12}\right)c^{4}-\left(4.49614\times 10^{-13}\right)d^{4}+\left(6.81304\times 10^{-8}\right)b^{3}-\;\left(9.42366\times 10^{-10}\right)c^{3}+\left(5.55359\times 10^{-10}\right)d^{3}+\left(1.45796\times 10^{-5}\right)b^{2}-\left(5.88341\times 10^{-7}\right)c^{2}+\;\left(1.14292\times 10^{-6}\right)d^{2}+\left(1.88691\times 10^{-3}\right)b-\left(1.57535\times 10^{-4}\right)c+\left(1.33691\times 10^{-3}\right)d+1.24475\times 10^{-1}$$

for GPBMs produced from the Catalagzi TPP FFA at 100 °C

(13) $$\mathrm{RA}\;\left(\%\right)=8.87011\times 10^{-1}-\left(1.47085\times 10^{-2}\right)a-\left(1.39286\times 10^{-5}\right)a^{2}+\left(5.91645\times 10^{-9}\right)a^{3}\;+\left(4.20831\times 10^{-11}\right)a^{4}+\left(1.07088\times 10^{-13}\right)a^{5}-\left(2.64208\times 10^{-3}\right)b-(6.1538\times 10^{-6})b^{2}\;+\left(1.18374\times 10^{-8}\right)b^{3}+\left(1.28551\times 10^{-10}\right)b^{4}+\left(5.33933\times 10^{-13}\right)b^{5}+\left(1.14801\times 10^{-3}\right)c\;+\left(1.12699\times 10^{-6}\right)c^{2}+\left(1.05019\times 10^{-9}\right)c^{3}+\left(9.8909\times 10^{-13}\right)c^{4}+\left(9.48881\times 10^{-16}\right)c^{5}\;+\left(9.94322\times 10^{-4}\right)d+\left(1.05721\times 10^{-6}\right)d^{2}+\left(1.0887\times 10^{-9}\right)d^{3}+\left(1.10331\times 10^{-12}\right)d^{4}\;+\left(1.11359\times 10^{-15}\right)d^{5}$$

for GPBMs produced from the Isken TPP FFA at 70 °C


(14) $$\mathrm{RA}\;\left(\%\right)=11.16+0.00582a+(3.06\times 10^{-11})\times a^{2}-1.6288\times 10^{-8}a^{3}-8.1628\times 10^{-11}a^{4}\;-2.8616\times 10^{-13}a^{5}+0.002b-4.7641\times 10^{-7}b^{2}-5.76532\times 10^{-9}b^{3}\;-1.53343\times 10^{-11}b^{4}-3.273\times 10^{-14}b^{5}+0.00133c+0.0000012c^{2}+1.00626\times 10^{-9}c^{3}\;+8.668211\times 10^{-13}c^{4}+7.734\times 10^{-16}c^{5}+0.00189d+0.00000140+4.8688\times 10^{-10}d^{3}\;-8.2528\times 10^{-13}d^{4}-2.516434\times 10^{-15}d^{5}$$

for GPBMs produced from the Isken TPP FFA at 100 °C

(15) $$\mathrm{RA}\;\left(\%\right)=3.53809+\left(6.06097\times 10^{-3}\right)a-\left(3.78929\times 10^{-6}\right)a^{2}-\left(3.65212\times 10^{-8}\right)a^{3}-\;\left(8.36025\times 10^{-11}\right)a^{4}+\left(7.68186\times 10^{-3}\right)-\left(6.65835\times 10^{-6}\right)b^{2}-\left(3.06929\times 10^{-8}\right)b^{3}-\;\left(5.32099\times 10^{-11}\right)b^{4}-\left(1.56723\times 10^{-14}\right)b^{5}+\left(2.01803\times 10^{-3}\right)c+\left(5.98923\times 10^{-7}\right)c^{2}-\;\left(6.45403\times 10^{-11}\right)c^{3}-\left(2.97268\times 10^{-13}\right)c^{4}-\left(3.5863\times 10^{-16}\right)c^{5}-\left(8.17871\times 10^{-3}\right)d-\;\left(5.39477\times 10^{-6}\right)d^{2}+\left(2.12577\times 10^{-10}\right)d^{3}+\left(8.61754\times 10^{-12}\right)d^{4}$$

In the above equations, a, b, c, and d are quantities of NaOH (g), Na_2_SiO_3_ (g), FFAs (g), and RILEM Cembureau sand (g), respectively. By calculating the optimum of mixture ingredients, the equations presented above were maximized to find the maximum of the RA. The SA and DE algorithms were applied separately considering upper and lower bounds expressed as follows:

(16) 0 ≤a≤400 g

(17) 0≤b≤400 g

(18) 0≤c≤1000 g

(19) 0≤d≤800 g

(20) 0≤a+b≤500 g

(21) 0≤c+d≤1200 g

The constraints of optimization in Equations (16)–(21) were obtained considering the maximum value of raw materials presented in Table 5. However, the amount of ingredients higher than this resulting from maximum values were chosen as upper constraints of optimization. Value 0 was fixed as the lower limit.

**Figure 3:**
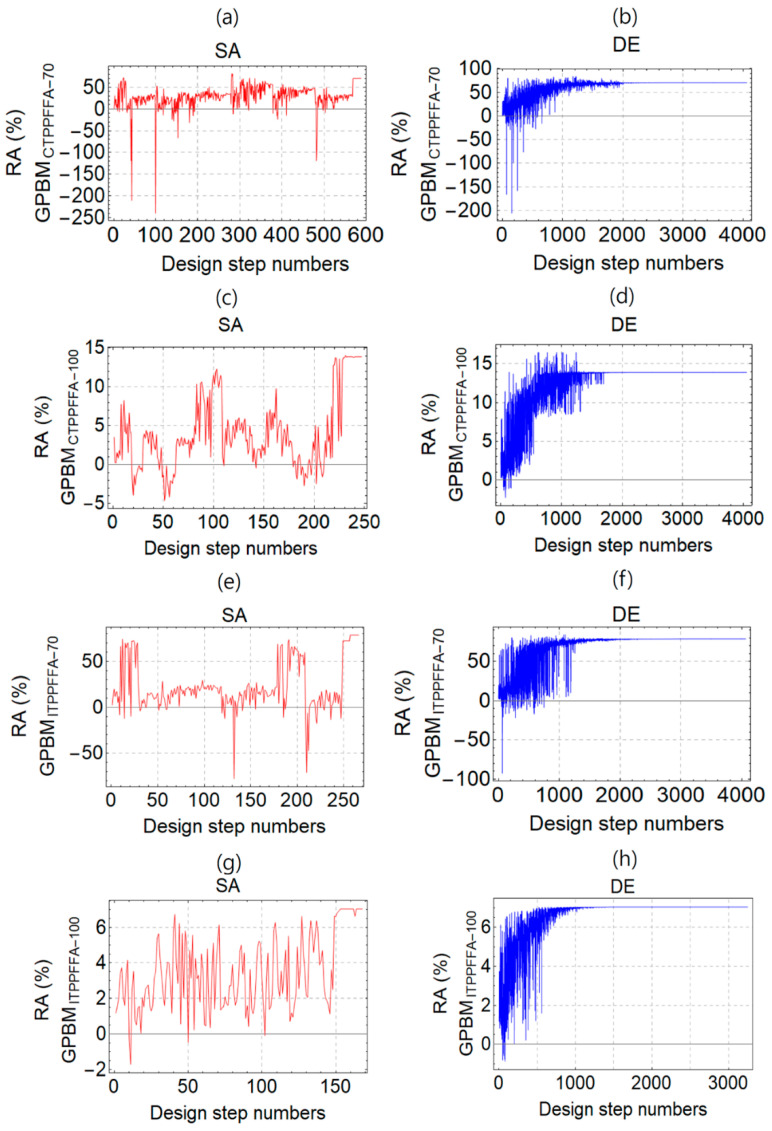
The process of radiation absorption (RA, %) optimization with respect to design step numbers by using the simulated annealing (SA), and the differential evolution (DE) methods. GPBMs were produced from the Catalagzi TPP FFAs and cured at temperatures of (**a**,**b**) 70 °C (GPBM_CTPPFFA-70_) and (**c**,**d**) 100 °C (GPBM_CTPPFFA-100_), as well as GPBM produced from the Isken TPP FFAs and cured at temperatures of (**e**,**f**) 70 °C (GPBM_ITPPFFA-100_) and (**g**,**h**) 100 °C (GPBM_ITPPFFA-100_).

Figure 3a–h depict the process of RA optimization with respect to design step numbers by using the SA and DE methods. The figures show how RA converges to a maximum during the optimization. The results are presented for the GPBMs produced from the Catalagzi and Isken TPP FFAs cured at 70 and 100 °C.

After the optimization process for GPBMs produced from the Catalagzi and Isken TPP FFAs at 70 °C (CTPP-70 and ITPP-70) and 100 °C (CTPP-100 and ITPP-100), the unknown coefficient of the predicted polynomial functions and optimum value of the RA % were calculated as presented in Table 6.

**Figure 4:**
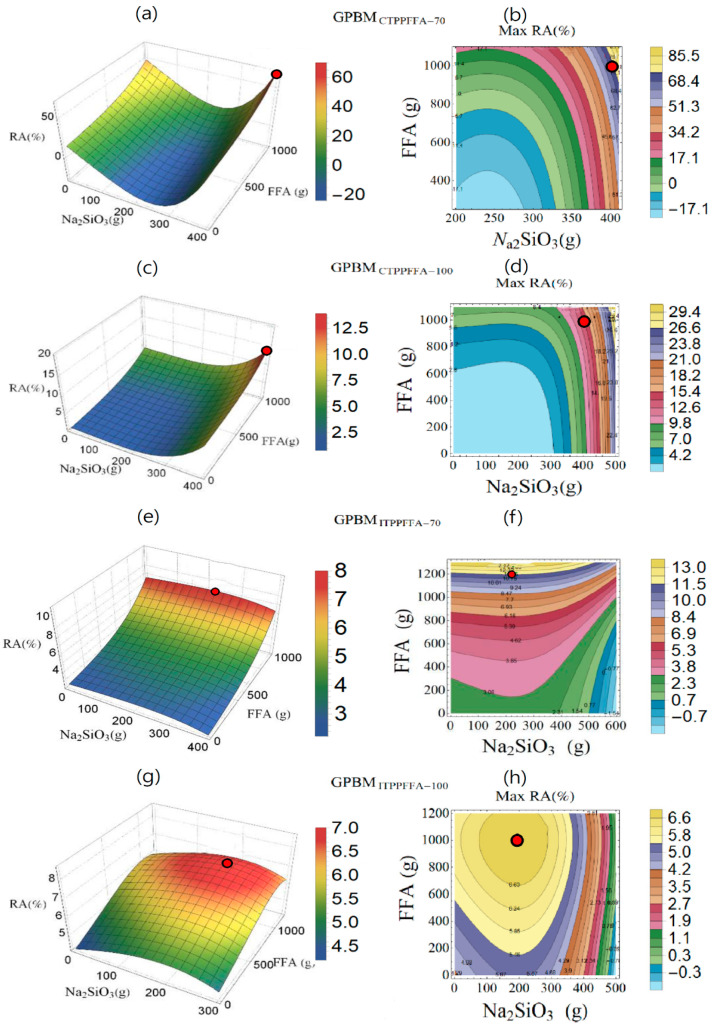
Variation of radiation absorption (RA, %) in three dimensions and contour plot graph with respect to Na_2_SiO_3_ and FFA quantities for GPBM produced from the Catalagzi TPP FFA at curing temperatures of (**a**,**b**) 70 °C (GPBM_CTPPFFA-70_), (**c**,**d**) 100 °C (GPBM_CTPPFFA-100_); and GPBM produced from the Isken TPP FFA at curing temperatures of (**e**,**f**) 70 °C (GPBM_ITPPFFA-70_), (**g**,**h**) 100 °C (GPBM_ITPPFFA-100_). Red dots indicate the optimum points.

The setting of ingredients for the production of GPBM based on the Catalagzi and Isken TPP FFAs and curing temperatures of 70 and 100 °C is shown in three dimensions and contour plots in Figure 4. They were calculated according to the maximum point and the variation of RA with respect to Na_2_SiO_3_ and FFA. The red circle points in Figure 4 showed variation and maximum RA (%). With an increasing amount of the Catalagzi TPP FFA in GPBM cured at 70 °C, an increase in RA was calculated at each amount of Na_2_SiO_3_ (Figure 4a,b). After the minimum point, the Na_2_SiO_3_ quantity caused a rise in RA. In the case of the GPBMs produced from the Catalagzi TPP FFA at 100 °C, the RA increased along with the increase in FFA and Na_2_SiO_3_ quantities (Figure 4c,d). For GPBMs produced from the Isken TPP FFA at 70 °C, an increase in FFA amount resulted in higher RA, while an increase in Na_2_SiO_3_ quantity led to RA decrease (Figure 4e,f). RA for the GPBMs produced from the Isken TPP FFA at 100 °C depended on defined quantities of FFA and Na_2_SiO_3_. Their improvement led to a rise in RA (Figure 4g,h). However, after the maximum point, the quantity of these variables caused a decrease in RA.

According to the mathematical model developed in this study, the effect of FFAs supplied from different TPPs on RA is an important issue.

## 4. Conclusions

The suitable features of GPBMs produced from the Catalagzi and Isken TPP FFAs consist of (i) use of raw materials (FFAs) and chemical activators (NaOH and/or Na_2_SiO_3_) instead of the cement, which accounts for a net reduction in energy use and greenhouse gas during the production; (ii) use of waste FFAs instead of the natural non-renewable sources; (iii) reuse of FFAs to reduce the storage of toxic waste; (iv) and increased life of building structures due to the improved material durability. With the above in mind and proving better engineering properties, we describe the developed GPBM material as an eco-geopolymer.

Using a newly designed measurement system consisting of the LRS cage, GPBMs were shown to have an excellent potential to serve as a barrier against harmful radiation. Mathematical modeling with the least-squares method was used to fit a polynomial function to quantities of FFAs, sand, NaOH, and Na_2_S_i_O_3_ and as a result find their optimum to achieve the maximum RA.

The detailed conclusions include:

(1) FFAs with total aggregate content of 70–80%, 12 M NaOH, the Na_2_SiO_3_/NaOH ratio of 1–2.5, and 24 h of curing at 70 or 100 °C all represent the conditions for GPBM production that result in final materials with an average compressive strength of 40–44 and 58–63 MPa for the GPBM produced from the Catalagzi TPP and Isken TPP FFAs, respectively.
(2) Higher reactivity of the Isken TPP FFA, and thus better mechanical and physical properties of the geopolymer, resulted from finer particles and greater surface area of raw material. The highest compressive strength was measured as 93.3 MPa for the GPBM produced with 10% NaOH and cured at 100 °C.
(3) The best GPBM (produced from the Isken TPP FFA) had the highest RA of 12.5%, density of 1.70 g cm^−3^, porosity of 19.9%, water absorption of 12.4%, and compressive strength of 57.3 MPa; thus, eco-friendly GPBMs are lightweight construction materials with good mechanical properties.
(4) According to the mathematical model developed in this study, the effect of FFA/alkali activator type and quantity on RA is an important issue. Optimization is required to obtain maximum RA values. Mathematical modeling and appropriate algorithms can provide this without costly experimentation.

However, the performance of GPBMs under radiation must be deeply understood. Radiation can lead to a change in the microstructure of a material and related chemical, physical, and mechanical parameters; thus, the dependences require further studies. Predicting GPBM properties and methodology with mathematical models seems irreplaceable, particularly for production of sophisticated materials for special purposes. Attempts have been made to produce materials such as radiation shielding concrete (RSC) [61] and ambient-cured heavyweight geopolymer concrete (HWGC) [62] to protect from the sources that emit harmful radiation in the medical and nuclear industries, and lunar building materials which need to meet criteria of resistance to severe temperature cycles (102.4  to 387.1 K), stability in a vacuum environment, minimal water requirements, and sourcing from local Moon materials [63].

## Figures and Tables

**Table 1:** GPBM samples produced based on the Catalagzi and Isken TPP FFAs, with or without the RILEM Cembureau standard sand, activated with the alkali activators (NaOH and/or Na_2_SiO_3_) in different proportions, i.e., NaOH used at a ratio of 10%, 15%, 25% alkaline activator to FFA (by weight) and NaOH/Na_2_SiO_3_ solutions (1:0, 1:1, 1:1.5, 1:2, and 1:2.5) used at a ratio of 10% and 20% alkaline activator to FFA (by weight).

FFA Type	NaOH to Na_2_SiO_3_ Ratio	Sand	Alkaline Solution to FFA (% by Weight)	Curing Temperature
Isken TPP, Catalagzi TPP	1:0	+	10%	70 °C, 100 °C
	1:0	-	15%, 25%	
	1:1	+	10%	
	1:1	-	10%	
	1:1.5	+	10%, 20%	
	1:1.5	-	10%, 20%	
	1:2	+	10%, 20%	
	1:2	-	10%, 20%	
	1:2.5	+	20%	
	1:2.5	-	20%	

**Table 2:** Chemical composition of oxides (%) and loss on ignition (LOI, %) determined for the Catalagzi and Isken TPP FFAs.

FFA Type	SiO_2_	Al_2_O_3_	Fe_2_O_3_	CaO	Na_2_O	MgO	K_2_O	SO_3_	Other Oxides	LOI
Catalagzi TPP	54.08	26.08	6.68	2.00	0.79	2.67	4.53	0.73	2.44	1.52
Isken TPP	62.28	21.46	7.01	1.53	0.26	2.37	3.81	0.07	1.21	1.78

**Table 3:** Physical properties of the Catalagzi and Isken TPP FFAs.

Properties	Catalagzi TPP FFA	Isken TPP FFA
BET (m^2^ g^−1^)	1.11	2.26
Specific gravity	2.04	2.25
Air-dried loose bulk density (g cm^−3^)	0.87	1.10
Air-dried tight bulk density (g cm^−3^)	1.04	1.14
Oven-dried loose bulk density (g cm^−3^)	0.75	0.98
Oven-dried tight bulk density (g cm^−3^)	0.88	1.05

**Table 4:** The RILEM Cembureau standard sand (the left panel) and its properties (table).

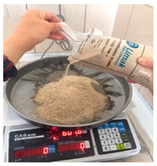	**Diameter of Sand Grain**	0.08	0.16	0.5	1.0	1.6	2.0
	**Remaining (%)**	99	87	72	34	6	0
	**Limits of specification (%)**	99 ± 1	99 ± 5	67 ± 5	33 ± 5	7 ± 5	0

**Table 5:** The material characteristic (density, porosity), mechanical (flexural and compressive strength), and physical (water absorption, radiation absorption) properties of the GPBMs produced based on FFAs supplied from the Catalagzi and Isken TPPs and alkali activators (NaOH and/or Na_2_SiO_3_) in different ratios.

	Mixing Ratio of Raw Materials			GPBM Properties					
Sample No	NaOH (g)	Na_2_SiO_3_ (g)	Alkaline Solution to FFA (% by Weight)	Density (g cm^−3^)	Porosity (%)	Water Absorption (%)	Flexural Strength (MPa)	Compressive Strength (MPa)	Radiation Absorption (%)
**GPBMs produced from Catalagzi TPP FFA**									
Curing temperature 70 °C									
1	300	-	15*	1.46	30.20	22.30	5.3	30.3	3.00
2	160	320	20	1.84	28.94	17.98	6.1	33.5	12.36
3	120	120	10	1.69	23.65	14.81	7.8	39.0	2.56
4	80	160	10	1.79	25.42	16.38	8.7	47.3	2.12
5	134	266	20*	1.46	21.91	15.63	4.6	53.0	2.38
Curing temperature 100 °C									
6	96	144	10	1.56	25.23	16.56	8.2	34.2	2.56
7	80	160	10	1.53	20.45	13.29	8.9	40.8	2.20
8	160	320	20	1.58	25.23	16.56	3.9	46.1	3.88
9	500	-	25*	1.45	31.23	23.75	3.9	47.0	2.03
10	160	240	20*	1.24	23.40	17.50	3.0	47.5	3.53
11	114	286	20*	1.32	22.80	17.46	8.8	49.0	5.21
**GPBMs produced from Isken TPP FFA**									
Curing temperature 70 °C									
12	174	346	20	1.94	28.94	17.98	13.1	46.9	0.88
13	500	-	25*	1.69	28.85	18.34	1.50	47.0	3.35
14	330	-	15*	1.81	26.69	16.46	7.35	63.5	5.91
15	125	315	20*	1.84	23.83	14.80	6.90	66.6	7.24
16	130	130	10	2.00	26.84	17.55	13.4	71.4	5.21
17	80	160	10	2.04	18.92	9.94	18.3	81.4	4.24
Curing temperature 100 °C									
18	110	110	10*	1.67	25.64	16.57	6.1	37.1	6.71
19	148	372	20	1.98	16.16	10.07	4.2	46.6	0.61
20	177	351	20	1.97	17.94	9.72	13.3	49.6	4.85
21	330	-	15*	1.69	23.09	14.45	16.2	52.4	4.68
22	125	315	20*	1.70	19.91	12.39	3.0	57.3	12.54
23	87	173	10	1.95	20.88	13.24	6.8	60.1	2.12
24	106	158	10	1.92	16.48	8.44	16.7	65.5	10.15
25	264	-	10	2.01	13.94	7.09	16.5	93.3	4.77

* GPBMs not containing RILEM Cembureau standard sand; other materials containing the sand at a ratio of FFA/sand = 1:1.

**Table 6:** Optimum values of the ingredients and calculated RA (%) for different GPBMs produced from the Catalagzi and Isken TPP FFAs at curing temperature 70 °C (GPBM_CTPP-70_ and GPBM_ITPP-70_) and 100 °C (GPBM_CTPP-100_ and GPBM_ITPP-100_) with respect to SA and DE algorithm.

Optimization Method *	GPBMs	N_a_OH (g)	Na_2_SiO_3_ (g)	FFA (g)	Standard Sand (g)	RA (%)
SA DE	GPBM_CTPP-70_	0	400	1000	200	70.7
	GPBM_CTPP-100_	0	400	1000	200	13.9
	GPBM_ITPP-70_	202	222	1000	200	8.0
	GPBM_ITPP-100_	169	194	1000	200	7.0

* Both methods (SA and DE) gave the same results.

## Data Availability

No new data were created or analyzed in this study. Data sharing is not applicable to this article.

## References

[1] Okoye F.N., Durgaprasad J., Singh N.B. (2016). Effect of silica fume on the mechanical properties of fly ash based-geopolymer concrete. Ceram. Int..

[2] Korniejenko K., Łach M., Dogan-Saglamtımur N., Furtos G., Mikuła J. (2020). The overview of mechanical properties of short natural fiber reinforced geopolymer composites. Environ. Res. Technol..

[3] Luhar S., Luhar I. (2020). Fly ash based geopolymer mortar-strength performance. Int. J. Recent Technol. Eng. (IJRTE).

[4] Szechyńska-Hebda M., Marczyk J., Ziejewska C., Hordyńska N., Mikuła J., Hebda M. (2019). Neutral geopolymer foams reinforced with cellulose studied with the FT-Raman spectroscopy. IOP Conf. Ser. Mater. Sci. Eng..

[5] Doğan-Sağlamtimur N., Bilgil A., Öztürk B. (2018). Reusability of Ashes for the Building Sector to Strengthen the Sustainability of Waste Management. Handbook of Research on Supply Chain Management for Sustainable Development.

[6] Kurtoğlu A.E., Alzeebaree R., Aljumaili O., Niş A., Gülşan M.E., Humur G., Çevik A. (2018). Mechanical and durability properties of fly ash and slag based geopolymer concrete. Adv. Concr. Constr..

[7] Huseien G.F., Mirza J., Ismail M., Ghoshal S.K., Hussein A.A. (2017). Geopolymer mortars as sustainable repair material: A comprehensive review. Renew. Sust. Energ. Rev..

[8] Gado R.A., Hebda M., Łach M., Mikuła J. (2020). Alkali Activation of Waste Clay Bricks: Influence of The Silica Modulus, SiO_2_/Na_2_O, H_2_O/Na_2_O Molar Ratio, and Liquid/Solid Ratio. Materials.

[9] Ren B., Zhao Y., Bai H., Kang S., Zhang T., Song S. (2021). Eco-friendly geopolymer prepared from solid wastes: A critical review. Chemosphere.

[10] Doğan-Sağlamtimur N., Bilgil A., Szechyńska-Hebda M., Parzych S., Hebda M. (2021). Eco-Friendly Fired Brick Produced from Industrial Ash and Natural Clay: A Study of Waste Reuse. Materials.

[11] Zhu Y. (2022). Zheng, Z.; Deng, Y.; Shi, C.; Zhang, Z. Advances in immobilization of radionuclide wastes by alkali activated cement and related materials. Cem. Concr. Comp..

[12] Asim N., Alghoul M., Mohammad M., Amin M.H., Akhtaruzzaman M., Amin N., Sopian K. (2019). Emerging sustainable solutions for depollution: Geopolymers. Constr. Build. Mater..

[13] Szechyńska-Hebda M., Marczyk J., Ziejewska C., Hordyńska N., Mikuła J., Hebda M. (2019). Optimal Design of pH-neutral Geopolymer Foams for Their Use in Ecological Plant Cultivation Systems. Materials.

[14] Davidovits J. (1979). SPE PACTEC’79.

[15] Koumoto T. (2019). Production of high compressive strength geopolymers considering fly ash or slag chemical composition. J. Mater. Civ. Eng..

[16] Djobo J.N.Y., Elimbi A., Tchakouté H.K., Kumar S. (2017). Volcanic ash-based geopolymer cements/concretes: The current state of the art and perpectives. Environ. Sci. Pollut. Res..

[17] Öz H.Ö., Doğan-Sağlamtimur N., Bilgil A., Tamer A., Günaydın K. (2021). Process Development of Fly Ash-Based Geopolymer Mortars in view of Mechanical Characteristics. Materials.

[18] Somna K., Jaturapitakkul C., Kajitvichyanukul P., Chindaprasirt P. (2011). NaOH-activated ground fly ash geopolymer cured at ambient temperature. Fuel.

[19] Arnoult M., Perronnet M., Autef A., Rossignol R. (2018). How to control the geopolymer setting time with the alkaline silicate solution. J. Non-Cryst Solids..

[20] Chindaprasirt P., De Silva P., Hanjitsuwan S. (2014). Effect of high-speed mixing on properties of high calcium fly ash geopolymer paste. Arab. J. Sci. Eng..

[21] Marczyk J., Ziejewska C., Gądek S., Korniejenko K., Łach M., Góra M., Kurek I., Doğan-Sağlamtimur N., Hebda M., Szechyńska-Hebda M. (2021). Hybrid Materials Based on Fly Ash, Metakaolin, and Cement for 3D Printing. Materials.

[22] Mohammed K.S., Azeez A.B., Al Bakri A.M.M., Hussin K., Rahmat A.B. (2014). The effect of barite content on anti radiation properties of geopolymer fly ash concrete incorporated natural rock ores of hematite. Int. J. Sci. Res..

[23] Aygün B. (2019). Neutron and gamma radiation shielding properties of high-temperature-resistant heavy concretes including chromite and wolframite. J. Radiat. Res. Appl. Sci..

[24] Akkurt I., Akyıldırım H., Mavi B., Kılınçarslan Ş., Basyigit C. (2012). The effect of pumice rate on the gamma absorption parameters of concrete. Acta Phys. Pol. A.

[25] Adewuyi Y.G. (2021). Recent Advances in Fly-Ash-Based Geopolymers: Potential on the Utilization for Sustainable Environmental Remediation. ACS Omega.

[26] Cantarel V., Motooka T., Yamagishi I. (2017). Geopolymers and Their Potential Applications in the Nuclear Waste Management Field-A Bibliographical Study-Japan Atomic Energy Agency.

[27] Jang J.G., Park S.M., Lee H.K. (2016). Physical barrier effect of geopolymeric waste form on diffusivity of cesium and strontium. J. Hazard. Mater..

[28] Kim B., Lee J., Kang J., Um W. (2021). Development of geopolymer waste form for immobilization of radioactive borate waste. J. Hazard. Mater..

[29] Shalbi S.M., Jaafar M.S., Ahmed N.M., Al-Jarrah A.M., Naji A., Alsadig Ahmed A., Qaeed M.A. (2017). Effect of fly ash geopolymer with 15% barium sulphate as a design shielding box on radiation attenuation using GafchramicXR-QA2 film dosimetry. IOSR J. Eng. (IOSRJEN).

[30] Kubissa W., Glinicki M.A., Dąbrowski M. (2018). Permeability testing of radiation shielding concrete manufactured at industrial scale. Mater. Struct..

[31] Basyigit C., Uysal V., Kilincarslan Ş., Mavi B., Günoğlu K., Akkurt I., Akkaş A. (2011). Investigating radiation shielding properties of different mineral origin heavyweight concretes. AIP Conf. Proc..

[32] The International Atomic Energy Agency (2013). The Behaviours of Cementitious Materials in Long Term Storage and Disposal of Radioactive Waste IAEA-TECDOC-1701.

[33] Tamzok N. (2017). Domestic Coal-based Thermal Power Plant. Potential, Constraints and Solutions, Thermal Power Plants in Turkey (Yerli Kömüre Dayalı Termik Santral Potansiyeli, Darboğazlar ve Çözüm Önerileri, Türkiye’de Termik Santraller).

[34] Özkan A., Turan E., Kaplan B.M. (2018). Investigation of fly ash effect on rheological and filtration properties of drilling muds. Fresenius Environ. Bull..

[35] TS EN 933-10 Tests for Geometrical Properties of Aggregates-Part 10: Assessment of Fines-Grading of Filler Aggregates (Air Jet Sieving), In Turkish Standards. https://standards.iteh.ai/catalog/standards/cen/cc9acb3e-3176-4461-8e2d-6cb1dfc816e7/en-933-10-2009.

[36] Arıcı E., Çelik E., Keleştemur O. (2021). An analysis of the engineering properties of mortars containing corn cob ash and polypropylene fiber using the Taguchi and Taguchi-based Grey Relational Analysis methods. Case Stud. Constr. Mater..

[37] ASTM C1437-15 Standard Test Method for Flow of Hydraulic Cement Mortar. In ASTM International. https://www.astm.org/c1437-15.html.

[38] TS EN 772-3 Methods of Test for Masonry Units-Part 3: Determination of Net Volume and Percentage of Voids of Clay Masonry Units by Hydrostatic Weighing; Türk Standardları Enstitüsü (Turkish Standards Institution): Ankara, Turkey.

[39] ASTM C348-14 Standard Test Method for Flexural Strength of Hydraulic-Cement Mortars. In ASTM International. https://www.astm.org/c0348-14.html.

[40] ASTM C349-14 Standard Test Method for Compressive Strength of Hydraulic-Cement Mortars (Using Portions of Prisms Broken in Flexure). In ASTM International. https://www.astm.org/c0349-14.html.

[41] TS EN 12390-3 Testing Hardened Concrete-Part 3: Compressive Strength of Test Specimens. http://www.vota.com.tr/assets/ts-en-12390-3.pdf.

[42] Diaz E.I., Allouche E.N., Eklund S. (2010). Factors affecting the suitability of fly ash as source material for geopolymers. Fuel.

[43] Valentim B.V., Hower J.C. (2010). Influence of feed and sampling systems on element partitioning in Kentucky fly ash. Int J. Coal Geol..

[44] Łach M., Gado R.A., Marczyk J., Ziejewska C., Dogan-Saglamtimur N., Mikuła J., Szechyńska-Hebda M., Hebda M. (2021). Process design for a production of sustainable materials from post-production clay. Materials.

[45] ASTM C618 Standard Specification for Coal Fly Ash and Raw or Calcined Natural Pozzolan for Use as a Mineral Admixture in Concrete, In ASTM International. https://www.astm.org/c0618-00.html.

[46] Osholana T.S., Dludlu M.K., Oboirien B., Sadiku R. (2020). Enhanced reactivity of geopolymers produced from fluidized bed combustion bottom ash. S. Afr. J. Chem. Eng..

[47] Bhatt A., Priyadarshini S., Mohanakrishnan A.A., Abri A., Sattler M., Techapaphawit S. (2019). Physical, chemical, and geotechnical properties of coal fly ash: A global review. Case Stud. Constr. Mater..

[48] TS EN 196-1 Methods of Testing Cement-Part 1: Determination of Strength, In Turkish Standards. https://standards.iteh.ai/catalog/standards/cen/37b8816e-4085-4dcc-a642-a383d9bddd6c/en-196-1-2016.

[49] Nath S.K., Kumar S. (2020). Role of particle fineness on engineering properties and microstructure of fly ash derived geopolymer. Constr. Build. Mater..

[50] Firdaus Y.İ., Daud R. (2017). Contribution of fineness level of fly ash to the compressive strength of geopolymer mortar. MATEC Web Conf..

[51] Jamkar S.S., Ghugal Y.M., Patankar S.V. (2013). Effect of fineness of fly ash fineness on workability and compressive strength of geopolymer concrete. Indian Concr. J..

[52] Cong P., Cheng Y. (2021). Advances in geopolymer materials: A comprehensive review. J. Traffic Transp. Eng..

[53] Demirel Y. (2015). An experimental purpose for correlation of data of rebound hammer as to axial load levels on the different strength of concrete columns. Düzce Üniversitesi Bilim ve Teknol. Dergisi..

[54] Yalcin M. (2021). Building Material/Application; Eskisehir, Turkey. https://insaat.eskisehir.edu.tr/muhsiny/MLZ204/icerik/12H-2)%20Beton%20üretimi-taze%20beton%20deneyleri-2-1%20(2020-2021).pdf.

[55] Mermerdaş K., Manguri S., Nassani D.E., Oleiwi S.M. (2017). Effect of aggregate properties on the mechanical and absorption characteristics of geopolymer mortar. Eng. Sci. Technol. Int. J..

[56] Mierzwiński D., Łach M., Hebda M., Walter J., Szechyńska-Hebda M., Mikuła J. (2019). Thermal phenomena of alkali-activated metakaolin studied with a negative temperature coefficient system. J. Therm Anal. Calorim..

[57] Korniejenko K., Figiela B., Miernik K., Ziejewska C., Marczyk J., Hebda M., Cheng A., Lin W.-T. (2021). Mechanical and Fracture Properties of Long Fiber Reinforced Geopolymer Composites. Materials.

[58] Leay L., Potts A., Donoclift T. (2018). Geopolymers from fly ash and their gamma irradiation. Mater. Lett..

[59] Uzun I. (2011). Numerical Analysis (in Turkish).

[60] Champion B., Strzebonski A. (2008). Constrained Optimization.

[61] Tyagi G., Singhal A., Routroy S., Bhunia D., Lahoti M. (2021). Radiation Shielding Concrete with alternate constituents: An approach to address multiple hazards. J. Hazard. Mater..

[62] Wang K., Tang Q., Cui X., He Y., Liu L. (2016). Development of near-zero water consumption cement materials via the geopolymerization of tektites and its implication for lunar construction. Sci. Rep.-UK.

[63] Montes C., Broussard K., Gongre M., Simicevic N., Mejia J., Tham J., Allouche E., Davis G. (2015). Evaluation of lunar regolith geopolymer binder as a radioactive shielding material for space exploration applications. Adv. Space Res..

